# lncRNA GAS5 Sensitizes Breast Cancer Cells to Ionizing Radiation by Inhibiting DNA Repair

**DOI:** 10.1155/2022/1987519

**Published:** 2022-01-11

**Authors:** Yan Ma, Lei Yu, Wenxing Yan, Ling Qiu, Jianqiu Zhang, Xiaojing Jia

**Affiliations:** Department of Radiation Oncology, The Second Hospital of Jilin University, Changchun, China 130021

## Abstract

Radioresistance of breast cancer is a major reason for therapeutic failure and limits further increases in the dose of radiation due to severe adverse effects. Recently, long noncoding RNAs (lncRNAs) have been shown to regulate cancer proliferation, chemoresistance, and radioresistance. Among these lncRNAs, lncRNA GAS5 expression was shown to be downregulated in breast cancer and related to trastuzumab resistance. However, its role in the radiation response is unclear. In this study, we demonstrated that lncRNA GAS5 expression was reduced in irradiated cells and that overexpression of GAS5 reduced cell viability and promoted cell apoptosis after irradiation. Moreover, overexpression of GAS5 resulted in increased G2/M arrest and unrepaired DNA damage, indicating a radiosensitizing role of GAS5 in breast cancer cells. Finally, we found that a GAS5-interacting miRNA, miR-21, reversed the radiosensitizing effects of GAS5 by inhibiting the apoptotic pathway. In conclusion, we found that lncRNA GAS5 sensitized breast cancer cells to ionizing radiation by inhibiting DNA repair and suppressing miR-21, identifying novel targets for breast cancer radiosensitization.

## 1. Introduction

Breast cancer is the most common cancer among women and remains a leading threat for patients [1]. In addition to surgery and chemotherapy, radiotherapy (RT) has been used to treat many patients and was found to reduce recurrence and mortality rates in breast cancer [2]. However, during radiotherapy, cancer cells become more radioresistant, which is a major reason for treatment failure as well as recurrence [2, 3]. Although radiotherapy is usually applied together with chemotherapeutic drugs as well as other strategies, breast cancer radioresistance remains a critical unsolved problem. Identification of novel effective targets for radiosensitization is urgently needed.

Long noncoding RNAs (lncRNAs) are a class of newly identified noncoding transcripts with a length of more than 200 nucleotides [4]. Several lncRNAs have been proven to participate in the regulation of proliferation and metastasis in breast cancer [5, 6]. Among all these molecules, lncRNA GAS5 expression was proven to be downregulated in breast cancer and was found to be related to trastuzumab resistance [7]. lncRNA GAS5 was also found to mediate the regulatory effects of Notch signaling on breast cancer cells [8, 9]. In addition to its role in breast cancer, lncRNA GAS5 was also found to have key roles in regulating cell apoptosis and chemoresistance in cervical cancer, liver cancer, and gastric cancer [10–12]. However, whether lncRNA GAS5 is involved in radiotherapy is unclear. In this study, we found that lncRNA GAS expression was lower in breast cancer cells, and overexpression of GAS5 effectively enhanced the radiosensitivity of breast cancer cell lines.

## 2. Materials and Methods

### 2.1. Cells and Treatments

Human breast cancer MCF-7 and MDA231 cells and normal MCF-10A cells were purchased from ATCC (USA) and maintained in our laboratory. All cells were cultured in DMEM with 10% fetal bovine serum (Gibco) in a 37°C humidified incubator under 5% CO_2_. The medium contained penicillin (100 units/ml) and streptomycin (100 *μ*g/ml). All experiments were approved by the ethical committee of the Second Hospital of Jilin University.

### 2.2. Irradiation and Transfection

Cells seeded in 25 cm^2^ flasks or 6-well plates (70% confluent) were exposed to different doses of ionizing radiation at a dose rate of 1 Gy/min with an X-ray linear accelerator (LINAC) in our hospital. For transfection, cells were seeded in 6-well plates at a concentration of 3 × 10^5^ cells/well. Twenty-four hours later, the cells were transfected with the lncRNA GAS5 overexpression plasmid or miR-21 mimics as well as NC mimics (Sangon Co., Shanghai) by using Lipofectamine 3000 reagent (Invitrogen).

### 2.3. RNA Extraction and Real-Time PCR Assay

Total RNA was extracted from cells by using TRIzol reagent (Sigma, US) according to the manufacturer's instructions. The concentration of RNA was measured with an ultraviolet spectrophotometer (GeneQuant 1300) in our laboratory, and RNA quality was ensured when A260/A280 > 2. Then, a cDNA synthesis kit (TaKaRa, Japan) was used to reverse transcribe the cDNA library with a 2 *μ*g RNA template. Real-time PCR was performed by using a SYBR Green Real-Time PCR Kit (TaKaRa, Japan) on an ABI 7500 machine. All reactions were performed according to the manufacturer's instructions. For determination of the expression of GAS5, the following primers were used [13]: forward, 5′-TGGTTCTGCTCCTGGTAACG-3′, reverse 5′-AGGATAACAGGTCTGCCTGC-3′; for GAPDH: forward 5′-GTCAAGGCTGAGAACGGGAA-3′, reverse 5′-AAATGAGCCCCAGCCTTCTC-3′. The primers were designed with the primer design tool at the NCBI database (https://www.ncbi.nlm.nih.gov/tools/primer-blast/).

### 2.4. CCK-8 Assay

For determination of cell viability after irradiation and different treatments, cells were seeded in 96-well plates at a concentration of 5000 cells per well. Cell viability was determined with a CCK-8 kit (Keygen). Briefly, after 24 h, the cells were treated with irradiation. Twenty-four or 48 h later, CCK-8 solution was added to the medium and incubated for 4 h. Then, the OD450 value was read with a Cytation 3 multimode reader from BioTek.

### 2.5. Apoptosis and Cell Cycle Assay

Twenty-four hours later, cell apoptosis was detected by using an Annexin V-FITC and PI double staining kit according to the manufacturer's instructions (Beyotime). Briefly, 10*E*6 cells were preincubated with Annexin V for 20 min, and PI was added immediately before sampling. Then, the cells were subjected to flow cytometric analysis (Beckman Coulter and the attached software). The cell cycle distribution was also assessed by flow cytometry as described previously [13].

### 2.6. Western Blot

Protein was extracted from cells by using RIPA extraction buffer (Thermo Scientific) on ice for 30 min according to the manufacturer's instructions. The obtained supernatant was separated with a 10% SDS-PAGE gel and transferred to PVDF membranes. Then, the membrane was blocked with 15% milk for 1 h and incubated with primary antibodies against Bax (Cell Signaling Tech., 1 : 1000), caspase 3 (Cell Signaling Tech., 1 : 1000), and beta-actin (Protein Tech., 1 : 1000). After incubation with secondary antibody, protein signals were detected with a chemiluminescence kit (Thermo Fisher Scientific, Inc.). Pictures were captured with an automatic image scanning system (Gene, Co.).

### 2.7. Comet DNA Damage Assay

DNA damage in cells after irradiation was detected by using a commercial comet assay kit (Trevigen, USA) according to the manufacturer's instructions. Briefly, cells molten in LMAgarose were mounted onto slides. After the slides were treated with alkaline solution, electrophoresis was performed at 25 V for 30 min in TBE buffer. Tail moments were analyzed with CASP software to determine the extent of DNA damage.

### 2.8. Statistical Analysis

The statistical analysis was performed by using GraphPad Prism 8 software according to the instructions. The data were analyzed by two-way ANOVA followed by Student's *t*-test. *P* < 0.05 was considered to be significant. Data are expressed as the mean ± SEM. All experiments were independently repeated three times.

## 3. Results

### 3.1. lncRNA GAS5 Expression Was Downregulated in Breast Cancer Cells and Inhibited by IR

**Figure 1:**
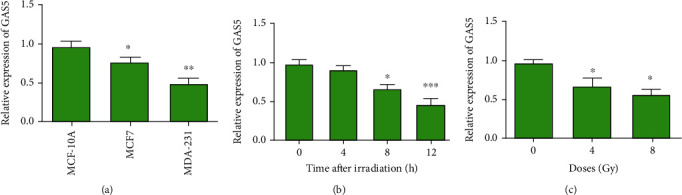
lncRNA GAS5 expression was downregulated in breast cancer cells and inhibited by IR. (a) Expression levels of lncRNA GAS5 in different breast cancer cell lines, including MCF-10A, MCF-7, and MDA-231. (b) Expression levels of lncRNA GAS5 in MCF-7 cells at different times (0, 4, 8, and 12 h) after 8 Gy irradiation. (c) Expression levels of lncRNA GAS5 in MCF-7 cells after 4 and 8 Gy irradiation at a dose rate of 1 Gy/min. ^∗^*P* < 0.05, ^∗∗^*P* < 0.01, and ^∗∗∗^*P* < 0.001 vs. the control group.

First, we assessed the expression of GAS5 in MCF-7 and MDA231 breast cancer cells and MCF-10A normal cells. We found that GAS5 expression was downregulated in the MCF7 and MDA231 cancer cells (Figure 1(a)). In response to IR, the GAS5 level also decreased in MCF-7 cells at 4 h and 8 h (Figure 1(b)). However, the downregulation of GAS5 expression was not dose-dependent, as it decreased to the same extent in the 4 Gy and 8 Gy groups (Figure 1(c)).

### 3.2. Overexpression of GAS5 Sensitized Cancer Cells to IR

**Figure 2:**
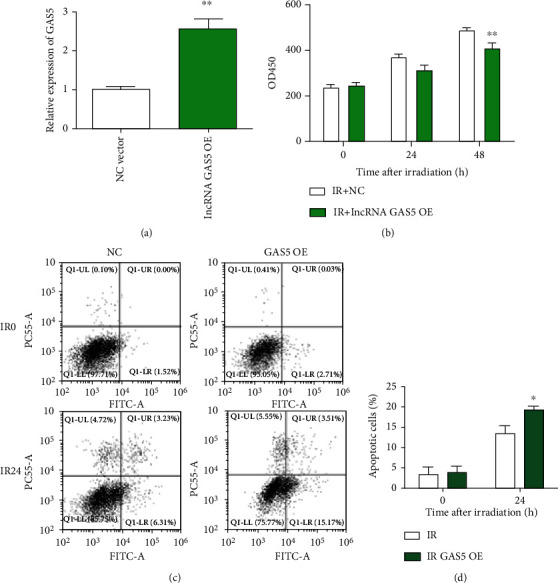
Overexpression of GAS5-sensitized cancer cells to IR. (a) Relative expression of GAS5 was detected by real-time PCR in the vector- and lncRNA GAS5-overexpressing MCF-7 cells at 48 h after transfection. (b) The viability of MCF-7 cells was determined with a CCK-8 kit at different times (0, 24, and 48 h) postirradiation (8 Gy). (c) Representative images of cell apoptosis were obtained with flow cytometry of MCF-7 cells with/without GAS5 and radiation treatments. (d) Quantitative analysis of cell apoptosis (upper right quadrant plus lower right quadrant) after different treatments. ^∗^*P* < 0.05 and ^∗∗^*P* < 0.01 vs. the control group.

As GAS5 expression was downregulated in irradiated breast cancer cells, we wanted to determine its role in the radiation response. We found that overexpression of GAS5 significantly suppressed the proliferation of MCF-7 cancer cells (Figures 2(a) and 2(b)). Then, we performed an apoptosis assay and found that radiation increased the apoptosis of the GAS5-overexpressing MCF-7 cells (Figures 2(c) and 2(d)).

### 3.3. Overexpression of GAS5 Resulted in Increased G2/M Cell Cycle Arrest and DNA Damage

**Figure 3:**
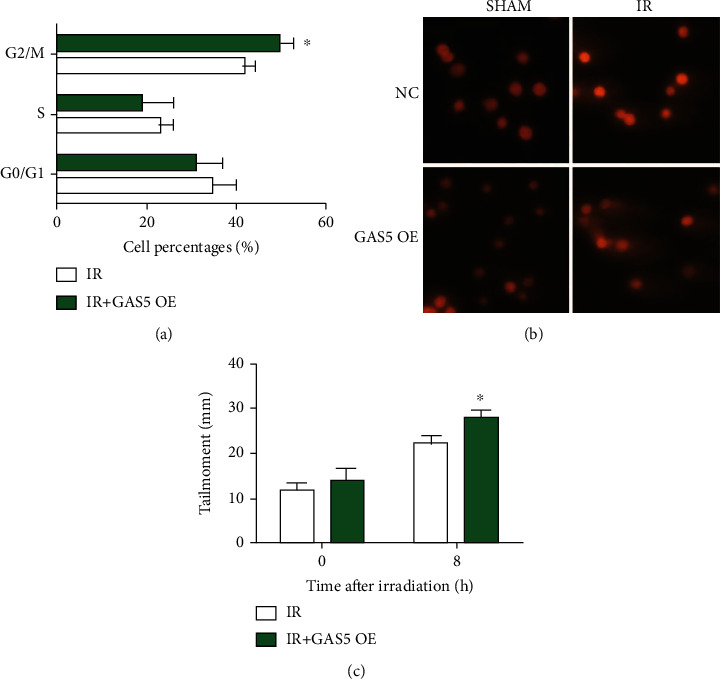
Overexpression of GAS5 resulted in increased G2/M cell cycle arrest and DNA damage. (a) The cell cycle distribution was analyzed by flow cytometric analysis of MCF-7 cells at 12 h after 8 Gy irradiation. (b) Representative images of the comet assay of the MCF-7 cells with 8 Gy irradiation and/or GAS5 overexpression. (c) Bar graph of the tail moment of the comet assay analyzed with CASP software in the cells from different groups. ^∗^*P* < 0.05 and ^∗∗^*P* < 0.01 vs. the control group.

IR induces DNA damage through direct and indirect effects, and thus, unrepaired DNA damage causes activation of cell cycle checkpoints and cell cycle arrest [14, 15]. In MCF-7 cells, we performed a cell cycle analysis and found that more cells arrested in the G2/M phase in the GAS5 overexpression group than in the single irradiation group (Figure 3(a)). The comet assay showed that more DNA damage remained unrepaired in the GAS5 group, indicating a role of GAS5 in cancer radiosensitization (Figures 3(b) and 3(c)).

### 3.4. GAS5 Regulated Radiosensitivity by Interacting with miR-21

**Figure 4:**
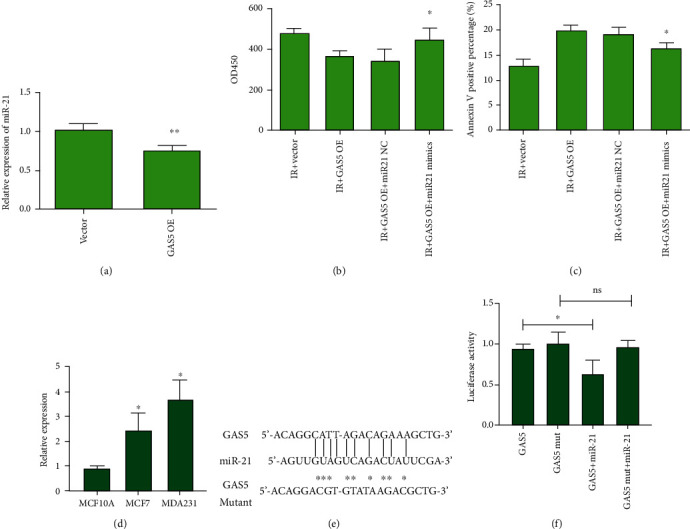
GAS5 regulated radiosensitivity by interacting with miR-21. (a) Relative expression level of miR-21 in the vector- and GAS5-overexpressing MCF-7 cells. (b, c) Analysis of cell viability and apoptosis of the MCF-7 irradiated cells (8 Gy) transfected with GAS5 and/or miR-21 mimics. For analysis of cell viability, CCK-8 assays were performed at 48 h. ^∗^*P* < 0.05 and ^∗∗^*P* < 0.01 vs. the GAS5-overexpressing group. (d) Expression of miR-21 in breast cancer cells was detected with real-time PCR assay. ^∗^*P* < 0.05 vs. that in MCF10A cells. (e) Sequence of wild-type and mutant GAS5 and seed sequence of miR-21 binding site. (f) Luciferase assay of lncRNA GAS5 and miR-21 cotransfected cells. ^∗^*P* < 0.05 vs. the GAS5 expression cells. ns means the comparison between miR-21 plus mutant GAS5 expression cells.

**Figure 5:**
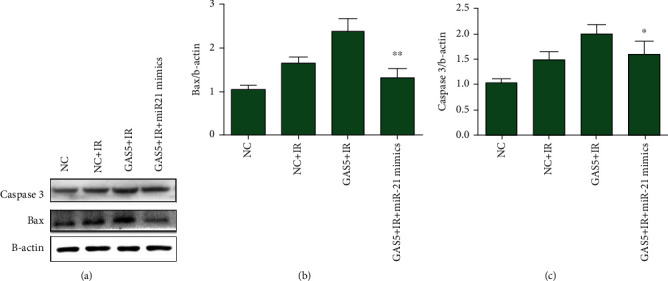
miR-21 inhibited the activation of the apoptosis pathway elevated by GAS5. (a) Representative images of Western blot assays detecting Bax, caspase 3, and beta-actin in extracts from the MCF-7 cells irradiated at a dose of 8 Gy. (b, c) Quantitative analysis of the raw density of Bax and caspase 3 compared to that of beta-actin with ImageJ software. ^∗^*P* < 0.05 and ^∗∗^*P* < 0.01 vs. the GAS5-overexpressing group.

lncRNA has an important role as a competing endogenous RNA (ceRNA) that binds microRNA, and in other cancer models, it was found that GAS5 directly interacts with miR-21, which accounts for radioresistance in lung cancers [16–18]. In this study, we found that miR-21 expression was downregulated in the GAS5-overexpressing MCF-7 cells (Figure 4(a)). miR-21 mimics rescued the inhibitory effects of GAS5 on cell proliferation and apoptosis after IR (Figures 4(b) and 4(c)). Through Western blot assays, we found that GAS5 overexpression increased the levels of Bax and caspase 3 compared with those of the single radiation group (Figures 5(a)–5(c)). In the GAS5 with miR-21 mimic-transfected group, the increases in Bax and caspase 3 expression were significantly inhibited (Figures 5(a)–5(c)), suggesting that miR-21 rescued the effects of GAS5 on cellular radiosensitivity.

## 4. Discussion

Instead of transcriptional noise, long noncoding RNAs have been proven to exert important functions in many cancers [4]. In addition to protein modification, posttranscriptional regulation also plays a critical role in cancer proliferation, metabolism, apoptosis, and the response to radiotherapy and chemotherapy. Among all lncRNAs, lncRNA GAS5 was shown to have downregulated expression in breast cancer tissues and cell lines and was closely correlated with cancer growth and chemoresistance [7, 19]. In this study, we investigated the effects of lncRNA GAS5 on cellular radiosensitivity and found that GAS5 could be a potential therapeutic target for treating breast cancer combined with IR.

First, we found that GAS5 expression was downregulated not only in breast cancer cells but also in irradiated cells, indicating a radiation-responsive role of GAS5. GAS5 overexpression also reduced cell viability and elevated cell apoptosis after exposure to IR. These data proved that GAS5 expression could sensitize cells to IR. The downregulation of GAS5 expression after irradiation also demonstrated the progressive radioresistance during the application of radiotherapy. DNA is the main target of IR, and radiation-induced cell death is also due to unrepaired DNA damage [20, 21]. We used a comet assay to assess DNA damage in GAS5-overexpressing cells, and we found that upregulated GAS5 expression resulted in more DNA damage after irradiation. This damage remained unrepaired and activated the cell cycle checkpoint [22, 23]; thus, we observed enhanced G2/M cell cycle arrest in irradiated cells. However, the underlying mechanism of the regulatory role of GAS5 in DNA damage and cell cycle regulation remains to be determined. We also found that GAS5 overexpression increased the activation of the apoptosis pathway, including Bax and caspase 3. miR-21 mimics inhibited the proapoptotic effects of GAS5.

One of the most important functions of lncRNAs is to sponge microRNAs as ceRNAs. Moreover, lncRNA GAS5 was shown to promote lymphangiogenesis by binding with miR-217 [24]. GAS5 also enhanced cholangiocarcinoma progression by regulating hsa-miR-1297 through direct binding [25]. In our study, we identified a potential interacting miRNA with GAS5, miR-21, and found that the GAS5-miR-21 axis contributed to the radiosensitivity of breast cancer cells. A previous report showed that GAS5 binds with miR-21 to regulate T cell function [26], which also supported our work. In contrast, miR-21 expression was found to be upregulated in breast cancer tissues and contributed to radiation resistance in breast cancer [27–29]. We found that a radiation-induced decrease in GAS5 expression, which also releases free miR-21, confers radioresistance. Overexpression of GAS5 resulted in downregulation of miR-21 expression, which increased radiosensitivity.

In conclusion, our findings identify lncRNA GAS5 as a potential target for breast cancer radiotherapy and show that it exerts a role in DNA repair and apoptotic regulation. The interaction between lncRNA GAS5 and miR-21 accounts for its radiosensitizing effects through regulation of the apoptosis pathway.

## Data Availability

All the data used to support the findings of this study are available from the corresponding author upon request.
